# Humans (really) are animals: picture-book reading influences 5-year-old urban children’s construal of the relation between humans and non-human animals

**DOI:** 10.3389/fpsyg.2014.00172

**Published:** 2014-03-17

**Authors:** Sandra R. Waxman, Patricia Herrmann, Jennie Woodring, Douglas L. Medin

**Affiliations:** Department of Psychology, Northwestern UniversityEvanston, IL, USA

**Keywords:** cognitive development, biological reasoning, cultural priming, children’s books, anthropocentrism

## Abstract

What is the relation between humans and non-human animals? From a *biological* perspective, we view humans as one species among many, but in the fables and films we create for children, we often offer an *anthropocentric* perspective, imbuing non-human animals with human-like characteristics. What are the consequences of these distinctly different perspectives on children’s reasoning about the natural world? Some have argued that children universally begin with an anthropocentric perspective and that acquiring a biological perspective requires a basic conceptual change (cf. Carey, 1985). But recent work reveals that this anthropocentric perspective, evidenced in urban 5-year-olds, is not evident in 3-year-olds (Herrmann etal., 2010). This indicates that the anthropocentric perspective is not an obligatory first step in children’s reasoning about biological phenomena. In the current paper, we introduced a priming manipulation to assess whether 5-year-olds’ reasoning about a novel biological property is influenced by the perspectives they encounter in children’s books. Just before participating in a reasoning task, each child read a book about bears with an experimenter. What varied was whether bears were depicted from an anthropomorphic (Berenstain Bears) or biological perspective (Animal Encyclopedia). The priming had a dramatic effect. Children reading the Berenstain Bears showed the standard anthropocentric reasoning pattern, but those reading the Animal Encyclopedia adopted a biological pattern. This offers evidence that urban 5-year-olds can adopt either a biological or a human-centered stance, depending upon the context. Thus, children’s books and other media are double-edged swords. Media may (inadvertently) support human-centered reasoning in young children, but may also be instrumental in redirecting children’s attention to a biological model.

## INTRODUCTION

Infants and young children greet the creatures of the natural world with special delight. For one of our daughters, it all started with her dog Roger – a stuffed animal who arrived in the newborn nursery only a few hours after she did and rarely left her side for more than a decade. Like most young children, she also delighted in images and animations of animals. Her first books included *Goodnight Moon* (whose main character is, after all, a little mouse). Years later, her favorite books included *Stellaluna* (a “switched at birth” story whose main character, a baby bat, finds herself living amongst a family of birds, all of whom talk – in English – about food preferences, emotions, and a sense of belonging). Perhaps not surprisingly, this little child who so loved animals announced that she was going to be a veterinarian when she grew up.

There is, of course, a huge gap between her storybook characters and the real, living and breathing animals that occupy the natural world. But is not this gap easily traversed? Do not the charming characters that young children encounter in their picture books support their natural fascination with animals and spark early learning about the biological world? These questions provide the underlying focus of this paper. Our goal is (a) to summarize evidence documenting how the relations between human and non-human animals are portrayed in children’s books, (b) to summarize recent research documenting how young children from diverse cultures reason about the relation between human and non-human animals, and (c) to present new experimental evidence documenting how the books that we read to children influence the ways in which they then reason about animals.

### CHILDREN’S PICTURE BOOKS

Picture books serve as sources of social engagement for children with adults in their close communities and as gateways for learning. By 15 months of age, infants successfully learn names (“vase,” “aardvark”) for novel objects that are introduced in picture books. More remarkably, infants spontaneously extend these names beyond pictorial representations, using them to name real three-dimensional objects when they encounter them in the world (Preissler and Carey, 2004; Ganea et al., 2008, 2011; Geraghty et al., 2011).

Although children’s books primarily have figured in research on early literacy and educational readiness (Poulsen et al., 1979; Pappas, 1986; Fletcher and Reese, 2005; Mar and Oatley, 2008), more recently this focus has been expanded to include investigations of children’s learning about the natural world (Ganea et al., 2008, 2011; Legare et al., 2013). Preschool-aged children can learn biological information presented in children’s books and use this information to reason about real, living animals (Ganea et al., 2008, 2011).

But children’s books provide something more than explicit information. They are cultural products that both *reflect* the orientations of their creators and may also *affect* the orientations adopted by their viewers (Morling and Lamoreaux, 2008; see Cole and Engeström, 1993 for an overview). For example, Tsai et al. (2007) identified two key differences in popular children’s books from the US and Taiwan. First, US storybooks were more likely than those from Taiwan to depict excited (versus calm) characters. Second, reading these books influenced the activity preferences and perceptions of happiness of children in both countries. Results like these indicate that children’s books reveal cultural orientations that affect *what* people think (D’Andrade, 1981) and *how* they think (Nisbett and Masuda, 2003, 2007).

Recent work from our research group provides converging evidence for the role of culture and cultural artifacts in development. As part of a larger project aimed at identifying how young children from different cultural communities reason about the natural world (e.g., Wolff et al., 1999; Waxman et al., 2007; Atran and Medin, 2008; Anggoro et al., 2010; Bang et al., 2010; Herrmann et al., 2010; Waxman et al., 2013; Medin and Bang, 2014), we asked whether and how our own perspectives of the natural world are embedded within children’s books. We examined popular children’s books that were written and illustrated by members of two cultural communities: Native Americans or non-Native Americans (Bang et al., in press; Dehghani et al., 2013). We found large cultural differences in the Native and non-Native books’ portrayals of the natural world and the place of humans within it. For example, illustrations from the Native-authored books provided a greater variety of perspectives and, most relevant to our present study, rarely if ever depicted animals wearing or surrounded by human artifacts, in sharp contrast to the heavily anthropomorphized non-Native books. Do these differences make a difference? That is, do children’s books also *shape* children’s reasoning about the natural world and their place within it?

### DEVELOPMENTAL MATTERS

This brings us to the question of how young children conceptualize and reason about the relation between human and non-human animals. As adults, we view this relation flexibly, adopting several different vantage points. Most Western-educated adults readily adopt a biological perspective, construing humans as one among the many species of the animal kingdom. But we also adopt a different construal, in which humans are set *apart from* the other animal species. Consider admonitions like “Don’t eat like an animal!” or the story of Genesis in which humans “⋯have dominion over the fish of the sea, and over the birds of the heavens, and over the cattle, and over all the earth, and over every creeping thing that creepeth upon the earth” (American Standard Version Bible, 1901) Even within the scientific community, humans are *apart from* non-human animals: Federal funding agencies require that research involving exclusively *human* participants be designated as research that does *not* include animals. Notice, however, that yet another perspective is pervasive, in which non-human animals are represented as surrogate humans. This strongly anthropocentric perspective is especially prevalent in the media designed for young children (cf., *Goodnight Moon, Stellaluna, Bambi*).

How do these different perspectives develop? Which are available early, before formal science instruction begins? One strong line of developmental work has suggested that when young children consider the natural world, they may be able to adopt only a single perspective, reasoning exclusively from an anthropocentric perspective and only later in childhood beginning to appreciate a biological perspective. But more recent work suggests that this picture might not be so clear.

### EARLY ANTHROPOCENTRIC REASONING

The strongest evidence for an early anthropocentric stance came from young urban children’s performance in an inductive reasoning task, pioneered by Carey (1985). In this task, participants were introduced to a novel biological property (e.g., “has an omentum”), told that this property is true of one biological kind (e.g., either a human or a dog), and then asked to decide which other entities might share this property. Carey documented a dramatic developmental progression, one that has been replicated robustly in several other urban communities. If the novel property was introduced as true of a human, 4-year olds projected the property broadly to other animals; but if the same property was attributed to a non-human animal (e.g., a dog), they did not generalize it broadly to other animals. In short, it was as if humans were the only proper base for generalization. Older children and adults projected the novel biological property broadly from one animal to another, whether it had been introduced as true of a human or non-human animal (e.g., a dog).

For decades, results like these were taken as evidence that young children begin reasoning about the biological world from an exclusively anthropocentric stance, comparing animals to a single prototype or standard (humans) and that they then undergo a conceptual change as they move from this human-centered model of *naïve psychology* (in which humans serve as the paragon) to the more mature, Western science-inspired model of *naïve biology* (in which humans are viewed as one biological kind among many; Carey, 1985, 1988, 1995).

This claim generated considerable interest and debate (Gelman and Wellman, 1991; Coley, 1995, 2007; Gutheil et al., 1998; Inagaki and Hatano, 2002; Heyman et al., 2003; Keil, 2007). Some have suggested that humans may be privileged in young children’s reasoning because urban children (who constitute the vast majority of research participants) simply know more about humans than non-human animals (Keil, 1992, 2007; Hatano and Inagaki, 1999; Heyman et al., 2003). Recent evidence from young children raised in rural communities, whose direct experience with non-human animals is considerably richer than that of urban-raised children, provided support for this interpretation. Rural 5-year-olds do not privilege humans over non-human animals when reasoning about biological phenomena (e.g., Sousa et al., 2002; Ross et al., 2003; Waxman and Medin, 2007). This outcome is important, but it does not shed light on whether anthropocentrism is the initial state. After all, because rural children have rich engagement with and exposure to the natural world, they may begin with an anthropocentric perspective but move beyond it sooner than their urban counterparts.

To address whether children really do universally begin reasoning from an anthropocentric perspective, we modified the now-classic induction task (Carey, 1985) to tap into the reasoning of children as young as 3 years of age (Herrmann et al., 2010). We reasoned that if the anthropocentric perspective is not an obligatory initial step but rather an acquired cultural model, then urban 3-year-olds might be *less* likely than their 5-year-old counterparts to privilege humans when reasoning about biological phenomena. The results provided clear support for this view: 3-year-old children showed no hint of anthropocentrism in their reasoning; they projected the novel biological property systematically from both human and non-human bases to other animals. Unlike 5-year-olds, 3-year-olds did not use humans as a privileged base for inductive reasoning about the biological world.

These developmental results also raised two important questions. First, if anthropocentrism is an acquired perspective, why is it acquired by 5-year-old children raised in some (cf. urban) but not all contexts? Second, what becomes of the biological perspective evidenced by 3-year-old children (Herrmann et al., 2010)? We suspect that this perspective is not discarded just 2 years later; instead, 5-year-old urban children may have access to both a biological perspective as well as an anthropocentric one (see also Gutheil et al., 1998). More specifically, we propose that in urban technologically saturated communities, where direct habitual contact with non-human animals is relatively limited (Rogoff et al., 2003), children encounter considerable support (intended or not) for an anthropocentric perspective and little in the way of direct experience to countervail it.

There is no doubt that images of non-human animals that children encounter in the books and media we design for them often take an anthropocentric cast (Marriott, 2002). But can representations like these actually influence their reasoning about the natural world?

## EXPERIMENT

To address this question, we selected two popular children’s books written and illustrated by European American authors with young children in mind. Both included bears as their focal character, but offered very different construals of bears. In one, *The Berenstain Bears’ Bedtime Battle *(Berenstain and Berenstain, 2004), bears are depicted as drawings, in a decidedly anthropocentric fashion (wearing clothes, speaking in English, engaging in human activities like birthday parties). In the other, *First Animal Encyclopedia* (Arlon, 2004), bears are depicted in a more realistic fashion, as photographs within their natural habitats and engaged in species-typical behaviors (foraging, building dens, caring for their young). If reading the Berenstain Bears book taps into an underlying anthropocentric model, then 5-year-old urban children reading excerpts from *Berenstain Bears* should adopt an anthropocentric stance in a subsequent reasoning task [privileging the human over the non-human animal (here, a dog) as an inductive base]. If reading about bears living in the natural world taps into a different, more biologically based construal, then 5-year-old urban children reading excerpts from the *Animal Encyclopedia* condition should adopt a different, non-anthropocentric stance (in which both humans and dogs serve as a strong inductive base for reasoning about other animals). Notice that this is a modest manipulation, especially when considered in light of the powerful media and conversational support that children receive for an anthropocentric perspective. If this book-reading manipulation is effective in eliciting biological patterns of reasoning even in the face of children’s saturation with anthopocentric images, this will suggest not only that children do indeed represent a biological perspective, but also can access it readily.

This design also allowed us to address another key question concerning the flexibility of children’s representations of the relation between human and non-human animals. If the anthropocentrism evinced by urban 5-year-olds is the only construal available to them when reasoning about the biological world, then reading *Animal Encyclopedia* should have little effect. But if they are also able to appreciate a non-anthropocentric model, and if *Animal Encyclopedia* effectively primes this model, they should be more likely to reveal a biological (rather than anthropocentric) pattern of reasoning after reading *Animal Encyclopedia*.

## METHODS

### PARTICIPANTS

Sixty-two typically developing 5-year-olds (34 female; 28 male), ranging from 60.1 to 71.5 months (*M* = 65.8), were recruited from the greater Chicago area and participated with their guardians’ consent. Children were drawn primarily from middle-class, majority-culture families. Two additional children were excluded from analysis for failure to meet inclusion criteria.

### MATERIALS

**FIGURE 1:**
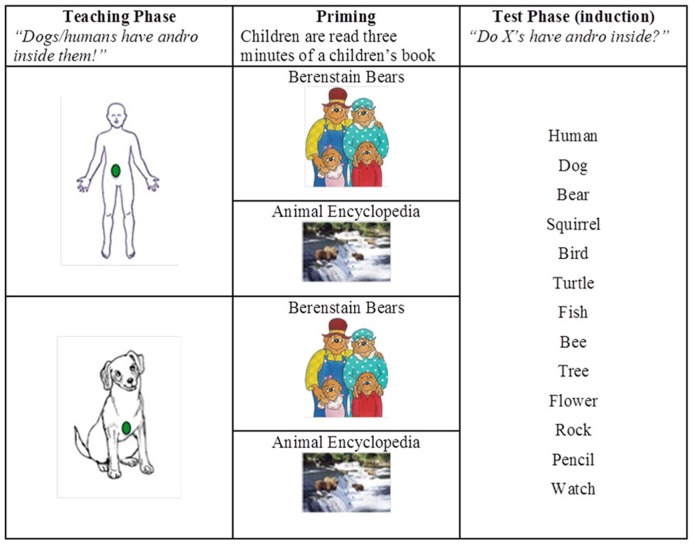
**Experimental design**. During the teaching phase, children were presented with either a human or dog; during the priming phase children were read to from Berenstain Bears or Animal Encyclopedia. All children were presented with all target pictures during the test phase.

Two children’s books, *The Berenstain Bears’ Bedtime Battle *(Berenstain and Berenstain, 2004) and *First Animal Encyclopedia* (Arlon, 2004) were used during the priming phase.**Both were written and illustrated with young audiences in mind. In *The Berenstain Bears*, the illustrations**were drawings; in *First Animal Encyclopedia, *the illustrations were photographic images. In addition, materials included (a) simple outline drawings of a human and a dog (used in the teaching phase), (b) six different finger-puppets (presented as pairs in the training and test phases), and (c) a series of 6” × 4” laminated, color photographs of humans, animals, plants, and artifacts, presented against natural backgrounds. Two photographs served as bases (human; dog). The remaining thirteen photographs served as targets. See **Figure 1**.

### PROCEDURE

Children sat across from the experimenter in a quiet testing room. Children were randomly assigned to either the human-base or dog-base condition during the teaching phase; within each base condition, children were randomly assigned to read either *Berenstain Bears* or *First Animal Encyclopedia* during the priming phase. The procedure involved four distinct phases: teaching, priming, training, and test. We use the dog-base to illustrate below.

#### Teaching phase

The child and experimenter each received a line drawing of the base (e.g., a dog). The experimenter introduced a novel biological property (e.g., “Dogs have *andro* inside them. *Andro* is roundish, greenish, and it goes inside!”). She then handed the child a crayon, saying, “Look! I’m drawing *andro* in my picture of a dog! Will you draw *andro* in yours?”

#### Priming phase

At this point, the experimenter read a few pages of either *Berenstain Bears* or *First Animal Encyclopedia* to the child. After three minutes, the experimenter closed the book and put it away.

#### Training phase

Next, the experimenter engaged the child in two training trials, designed to clarify the task for the child and to convey that sometimes the puppets were right, but that sometimes they were wrong. (This training phase was developed in Herrmann et al., 2010). The experimenter told the child that she had brought with her some pictures and some “silly puppets.” She explained that each puppet sometimes said the right thing, and sometimes was very silly, and that the child’s job was to help her (the experimenter) figure out which puppet was right. She then placed one puppet on either side of the child’s line drawing (e.g., dog) and initiated a brief puppet show, in which she posed questions and the puppets responded. To begin, she asked, “What do we have here?” One puppet asserted (correctly), “That’s a picture of a dog!”; the other countered (incorrectly), saying, “No. That’s not a picture of a dog!” The experimenter asked the child to decide which puppet was right (the first puppet) and to indicate their choice by pointing. Next, the puppets “spoke” again. This time, the first asserted (incorrectly), “That’s a picture of a chair!” and the second countered (correctly), “No! That’s not a picture of a chair!” Again, the child was instructed to point to the puppet that was correct (this time, the second puppet). If the child responded incorrectly, the experimenter repeated the puppet dialog and asked which puppet was right. If a child failed to respond correctly after three repetitions, the child was excluded from further analysis.

#### Test phase

To begin the test phase, the experimenter revealed all of the target photographs in random order, asking the child to identify each by name, and then providing feedback. She then shuffled the photographs and reminded the child, e.g., “Remember when we talked about *andro*? And we said that dogs (or people) have *andro* inside? Some other things have *andro* too. Let’s look”. She then introduced each target sequentially, in random order, with a finger puppet positioned on either side. For every question the experimenter posed (e.g., “What do you think? Do X’s have *andro* inside?”), one puppet answered in the affirmative (e.g., “Yes! X’s do have *andro* inside”) and the other countered in the negative (e.g., “No! X’s do not have *andro* inside”). The child’s task was to decide which puppet was right. Response-neutral encouragement was offered after any response (e.g., “Okay! Good for you!”). The experimenter then introduced another target, this time flanked by a different pair of puppets, and so on. The order in which the puppet pairs appeared and the order in which each “spoke” was counterbalanced. The experimenter recorded the child’s response to each target.

## RESULTS

**FIGURE 2:**
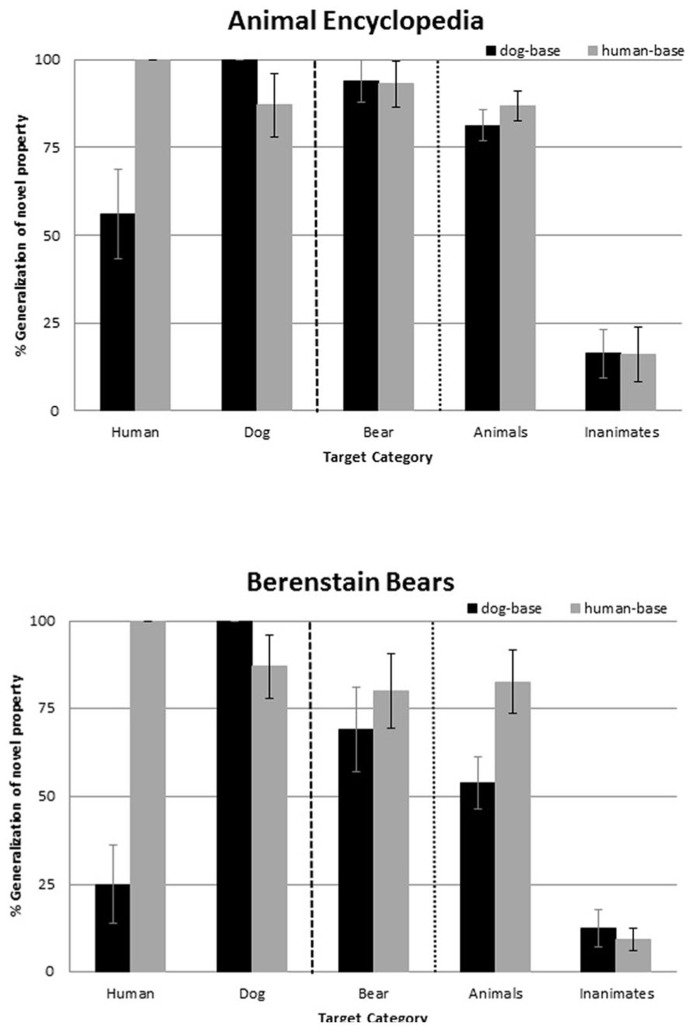
** Generalization to each target category, as a function of book and condition**. Error bars depict standard error of the mean.

The results, depicted in **Figure 2**, reveal that 5-year-old urban children responded to the distinctly different construals presented to them in the two books, and that these primes influenced their subsequent reasoning about a novel biological property. As predicted, children reading excerpts from *Berenstain Bears* showed the classic human-centered pattern, favoring humans over non-human animals as an inductive base. But those reading *Animal Encyclopedia* performed differently, providing no hint of the anthropocentric stance that, until now, has been considered the hallmark of their reasoning about the biological world.

We tailored our analyses to focus on three issues. For all analyses, *p* < 0.05 was set as the threshold for statistical significance. Moreover, the patterns exhibited by individual children converged with the mean patterns observed at each age.

### PROJECTING THE NOVEL BIOLOGICAL PROPERTY TO A NEW BEAR

First, we asked whether the way in which bears were represented in the book that they read influenced the likelihood that children would project a novel biological property to a new bear. To address this question, we considered children’s tendency to extend the novel biological property (learned in the teaching phase) to the *bear* test item. An ANOVA using Base (human- vs. dog-base) and Book (*Berenstain Bears* vs. *Animal Encyclopedia*) as between-participants factors revealed an effect for Book, *F*(1, 58) = 4.28, *p *< 0.05. Children reading *Animal Encyclopedia* (*M* = .94, SD = 0.07) were more likely than those reading *Berenstain Bears* (*M* = 0.74, SD = 0.07) to extend the novel property to the bear test item.

To provide a more direct test of our hypothesis, we conducted planned contrasts within each book. As predicted, for children reading either book, projections from the *human* to the bear were uniformly high [0.93 (SD = 0.26) and 0.80 (SD = 0.41) for *Berenstain Bears* and *Animal Encyclopedia*, respectively, *ns*], but their projections from the *dog* to the bear revealed an impact of the book that they had read: Here, children reading *Animal Encyclopedia* were more likely to extend the property from a *dog* to the new bear (*M* = 0.94, SD = 0.25) than were children reading the *Berenstain Bears* (*M* = 0.69, SD = 0.48), *p *< 0.05. Thus, children reading the *Berenstain Bears* showed an asymmetry that favored reasoning from humans (over dogs) as a base, but those reading the *Animal Encyclopedia* revealed no asymmetry.

This reveals that the perspective portrayed in the book was sufficiently strong to influence children’s tendency to extend a newly learned biological property to bears. Children who were primed with a book portraying bears realistically (as animals) adopted a biological stance, projecting the novel property from one animal (either a human or a dog) to the new bear presented at test. But children who were primed with a book portraying bears anthropomorphically adopted a human-centered reasoning pattern and were less likely to extend the novel property from one non-human animal (dog) to another (bear).

In the next analyses, we consider whether the book primes also influenced children’s expression of the two patterns – asymmetries and generalization patterns – that have been taken as signatures of reasoning from an anthropocentric perspective (Carey, 1985; Ross et al., 2003).

### ASYMMETRIES IN REASONING

Does the way in which bears were represented in the book that children read influence their tendency to project the novel biological property from *human* to *dog* and from *dog* to *human*? We predicted that children reading either book would be more likely to extend the property from a *human* to a *dog* than from a *dog *to a *human *(Carey, 1985; Herrmann et al., 2010), but that this asymmetry favoring humans would be less pronounced for children who had been primed with *Animal Encyclopedia* than *Berenstain Bears*. An ANOVA using Base (human- vs. dog-base) and Book (*Animal Encyclopedia* vs *Berenstain Bears*) as between-participants factors revealed a main effect for Base, *F*(1,58) = 18.30, *p *< 0.0001. Children were more likely to extend a novel property from a *human* to a *dog* (*M* = 0.87, SD = 0.35) than from a *dog* to a *human* (*M* = 0.41, SD = 0.50). This was mediated by an interaction between Base and Book, *F*(1,58) = 2.11, *p = *0.152, that fell short of statistical reliability but was consistent with the prediction that children primed with a biological construal (*Animal Encyclopedia*) would be less likely than those primed with an anthropocentric construal (*Berenstain Bears*) to favor humans over non-human animals (here, dog) in their reasoning.

We pursued this by conducting planned contrasts within each book. As predicted, children exposed to the anthropocentric book made significantly more projections from the *human* to the *dog* (*M* = 0.87, SD = 0.35) than from the *dog* to the *human* (*M *= 0.25, SD = 0.45), *p* < 0.05. This replicates the pattern reported in previous work with urban four- and 5-year-olds (Carey, 1985; Herrmann et al., 2010). But children exposed to the biological book revealed no such asymmetry, with no reliable difference in their projections from a *dog* to a *human* (*M* = 0.56, SD = 0.51) versus from a *human* to a *dog* (*M* = 0.87, SD = 0.35), *ns.*

### GENERALIZATION PATTERNS TO OTHER ANIMALS AND TO INANIMATE OBJECTS

Finally, we focused on children’s responses to the remaining targets, asking whether the way in which bears were represented in the book prime influenced their patterns of generalizing the novel biological property to other animals and to inanimate objects. We predicted that children reading either book would be more likely to generalize the property to other animals if it was introduced in conjunction with a *human* than a *dog* (Carey, 1985; Herrmann et al., 2010), but that this generalization pattern favoring humans would be less pronounced for children who had read the biologically oriented book than the anthropocentric book. For this analysis, any targets that were included in the previous analyses (bear, dog, human) were excluded. An ANOVA with Book (*Animal Encyclopedia* vs. *Berenstain Bears*) and Base (human-base vs. dog-base) as between-participant factors and Target category (animals vs. inanimates) as a within-participants factor revealed a main effect for Target category, *F*(1, 58) = 196.369, *p *= 0.000. Independent of the book they had read, children’s projections to other animals were uniformly high and their projections to the inanimates were uniformly low. This was qualified by an interaction between Target category and Base, *F*(1, 58) = 4.468, *p *< 0.05, as well as a main effect for Book, *F*(1,58) = 5.345, *p < *0.05: children reading *Animal Encyclopedia* were more likely than those reading *Berenstain Bears* to generalize the novel biological property. Moreover, as in the previous two analyses, the influence of the book prime was more pronounced for children reasoning from the *dog-* than from the *human-base*.

We pursued this by conducting planned comparisons of children’s generalization patterns within each book. As predicted, those reading the anthropocentric book revealed the classic anthropocentric pattern: they were more likely to extend the novel property to other animals if it had been introduced on a *human* (*M* = 0.83, SD = 0.36) than a *dog *(*M* = 0.54, SD = 0.30), *p *< 0.05. But children reading the biological book showed a different pattern: their results reveal no evidence that humans served as a privileged inductive base. Instead, their tendency to extend the novel property to other animals was comparable, whether it had been introduced in conjunction with the human (*M* = 0.87, SD = 0.16) or the dog (*M* = 0.81, SD = 0.17), *ns*.

In sum, children were indeed sensitive to the distinctly different construals of animals offered in these two children’s books, and this had consequences on their biological reasoning in a subsequent induction task. Children reading *Berenstain Bears* – a book filled with anthropomorphized images and information about bears – favored humans over non-human animals as an inductive base, replicating previous reports (Carey, 1985; Herrmann et al., 2010). In contrast, children reading *Animal Encyclopedia* – a book filled with realistic images and biological information about bears – revealed no anthropocentrism. Moreover, *Animal Encyclopedia* served as a more effective support for children’s learning about biological properties of a new bear than did *Berenstain Bears.*

## DISCUSSION

This experiment offers four insights into the influence of picture books in children’s developing notions of the natural world. First, the results reveal that 5-year-old children’s sensitivity to the representations of non-human animals in children’s books is keen enough to influence their reasoning. Children who were primed with a book portraying bears realistically (as animals) adopted a biological stance, projecting the novel property from one animal (either a human or a dog) to other animals at test. But children who were primed with a book portraying bears anthropomorphically adopted a human-centered reasoning pattern and were less likely to extend the novel property from one non-human animal (dog) to others. Second, these results provide unambiguous evidence that the anthropocentric pattern of reasoning *typically* observed in urban 5-year-old children on the category-based induction task is not the only perspective available to them in reasoning about the biological world. Instead, the perspective they adopt is influenced by the way in which non-human animals are represented in a children’s book they read moments earlier. Third, these results reveal that when we “humanize” non-human animals in our stories to young children, we do not promote learning about the biological world. Instead, anthropomorphizing non-human animals appears to have the opposite effect. This outcome is consistent with other recent work (Richert et al., 2009; Walker et al., 2012; Legare et al., 2013). Finally, these results have implications for promoting science learning in young children. If we understand the model(s) that children bring with them to their classrooms, we may be better able to promote their learning (Bang et al., 2007; National Research Council, 2007).

These results also provide insight into why anthropocentric patterns of reasoning about the biological world might emerge in urban 5-year-old children. We know that by 5 years of age, children are especially sensitive to cultural discourse about biological phenomena (Waxman et al., 2007). In urban communities, where direct contact with non-human animals is relatively limited (Rogoff et al., 2003) and where images of non-human animals in children’s books, discourse, and media often take an anthropocentric cast (Marriott, 2002; Pentimonti et al., 2011; Dehghani et al., 2013), young children encounter considerable support (intended or not) for an anthropocentric perspective. The results of the current experiment reveal their sensitivity to these anthropocentric portrayals in their reasoning. We suspect that in rural communities, where children’s engagement with the natural world is less mediated by artifacts, exposure to anthropocentric images may exert less impact on children’s developing notions of the biological world. A goal of our ongoing work is to ascertain whether rural children, or children from non-Western cultural communities, are less likely than their urban counterparts to adopt a human-centered perspective when exposed to anthropocentric media primes.

Another goal is to consider the impact of how animals are portrayed in other media designed for young children, extending the current results not only to other children’s books but also to films. Additional research will also be required to ascertain which features of these books (e.g., text, illustrations) – separately or in combination – were most influential in shaping children’s reasoning patterns and to discover how books written from the perspective of other cultural communities (c.f., Native American) might influence children’s reasoning about the natural world.

In closing, the experiment reported here reveals that priming with children’s books had a dramatic effect. Children primed with *Berenstain Bears* revealed the standard anthropocentric pattern. In contrast, children primed with *Animal Encyclopedia* adopted a biological reasoning pattern. This offers the first evidence of a distinctly biological reasoning pattern in urban 5-year-olds and suggests that they can move flexibly from a biological to a human-centered stance, depending upon the context at hand. Thus, children’s books and other media are double-edged swords. Media may (inadvertently) support human-centered reasoning in young children, but may also be instrumental in redirecting children’s attention to a biological model in which humans are one among the animal kinds.

## Conflict of Interest Statement

The authors declare that the research was conducted in the absence of any commercial or financial relationships that could be construed as a potential conflict of interest.
